# Improved pertussis vaccine uptake following in-hospital administration among pregnant women living with HIV

**DOI:** 10.1007/s00404-026-08323-4

**Published:** 2026-01-17

**Authors:** Sara Iannantuoni, Annunziata Carlea, Laura Letizia Mazzarelli, Luigi Falco, Oriana Imperatore, Dario Colacurci, Gennaro Esposito, Matteo Giudice, Concetta De Simone, Claudia Casella, Maria Rosaria Pagano, Carmen Buonaguro, Giuseppe Maria Maruotti, Maurizio Guida, Giuseppe Bifulco, Laura Sarno

**Affiliations:** 1 Department of Public Health, University of Naples Federico II, Naples, Italy; 2 Department of Neurosciences, Reproductive Science and Dentistry, University of Naples Federico II, Naples, Italy; 3 Mother and Child Department, University Hospital Federico II, Naples, Italy; 4 Department of Advanced Biomedical Science, University of Naples Federico II, Naples, Italy

**Keywords:** Vaccine uptake, Pertussis, Pregnant women living with HIV, Pregnancy, Vaccination

## Abstract

**Background:**

Pregnant women living with HIV (PWLHIV) are at increased risk of infectious complications during pregnancy, and HIV-exposed but uninfected women are more susceptible to serious infectious diseases. Therefore, maternal immunization during pregnancy is an essential standard of care for this patient population. However, vaccination during pregnancy is suboptimal among these patients.

**Methods:**

This is a single-center retrospective observational study, conducted at the University Hospital Federico II of Naples, Italy. We examined how our Center's vaccination uptake among PWLHIV changed after the introduction of in-hospital vaccine administration. To account for the small sample size and assess the robustness of the findings, Firth’s penalized logistic regression was performed as a sensitivity analysis.

**Results:**

Between January 2021 and December 2024, 41 PWLHIV have been referred to the Regional Referral Centre for HIV in Pregnancy. Out of 38 eligible patients, 21 received the Tdap vaccine, resulting in an overall uptake of 55.3%. Following the introduction of in-hospital vaccine administration in January 2023, we observed a significant increase in pertussis-containing vaccine uptake among PWLHIV. Vaccine uptake increased from 20% (January 2021–December 2022) to 78.3% after the introduction of in-hospital vaccine administration (January 2023–December 2024), (*p* < 0.001). The sensitivity analysis using Firth’s penalized logistic regression confirmed the independent association between in-hospital vaccination and vaccine uptake (adjusted OR 11.45; 95% CI 2.45–68.32; *p* < 0.001).

**Conclusions:**

We observed improved vaccine uptake after introducing vaccine administration within the hospital setting. We believe that this strategy might significantly improve vaccine administration among PWLHIV.

## What does this study add to the clinical work?

**Table:** 

Offering and administering Tdap directly within the antenatal HIV clinic — by the same obstetric team providing prenatal care — was associated with a marked increase in vaccine uptake among pregnant women living with HIV. Integrating in-hospital vaccination and repeated, structured counselling into routine visits can reduce missed opportunities and improve maternal immunization in this high-risk population.	

## Introduction

Pertussis is a respiratory infection caused by Bordetella pertussis that can lead to severe complications, particularly in infants during their first year of life [1]. In 2019, approximately 5.3 million children under 5 years of age died worldwide, and 21,7% of these deaths were due to vaccine-preventable diseases [2]. According to an Italian retrospective observational study, between 2007 and 2018, pertussis led to hospitalization in 77,9% of cases, and all pertussis-related deaths occurred in unvaccinated infants under 3 months [3]. To protect this vulnerable group, national health organizations in 58 countries have recommended maternal vaccination during the third trimester of pregnancy with the combined tetanus, diphtheria, and acellular pertussis-containing vaccine (Tdap) [4]. Maternal immunization has been demonstrated to be a safe and effective strategy. Vaccine effectiveness among infants under 3 months of age born to immunized mothers ranges from 69 to 91% for pertussis prevention, from 91 to 94% for hospitalization prevention, and is 95% for preventing related deaths [5]. While the vaccination coverage expected by 24 months of age, updated as of June 20, 2024, shows a coverage rate of 94,6% for the Tdap vaccine in Italy, data on maternal vaccinations in Italy are limited. The estimated maternal vaccination rate remains below 95% in most regions [6, 7], and vaccine uptake has declined further since the onset of the COVID-19 pandemic [8, 9], despite the introduction of maternal vaccination recommendations. As a consequence, an increase in pertussis cases was reported last year in several countries worldwide [8]. In Italy, a pertussis outbreak was recorded in 2024, with 108 hospitalizations and three deaths reported from January to 10 May 2024. Approximately 94% of mothers in the reported cases were unvaccinated, and 20% were not informed about Tdap vaccination during pregnancy [10]. These data highlight the importance of exploring strategies to improve vaccination during pregnancy [8, 11, 12].

Despite the availability of maternal vaccines and increasing global emphasis on immunization during pregnancy, pregnant women living with HIV (PWLHIV) remain a largely understudied population regarding vaccine uptake. Vaccine uptake during pregnancy among PWLHIV is significantly lower than in the general population of pregnant women. Studies [2] from high-income and low- and middle-income countries have consistently shown that PWLHIV have lower rates of vaccine uptake due to a combination of structural, clinical, and psychosocial barriers. Yet this population may benefit even more from maternal immunization strategies. A multicenter cohort study in the United States found that only 32.6% of PWLHIV received the Tdap vaccine during pregnancy [13]. Several factors, including healthcare access and socioeconomic barriers, may contribute to lower Tdap vaccination rates among PWLHIV. PWLHIV may face challenges in accessing consistent prenatal care, leading to missed opportunities for vaccination [13]. Therefore, despite the worldwide push to improve maternal immunization, PWLHIV remain a particularly vulnerable subgroup. Moreover, concerns about vaccine safety and efficacy, particularly among immunocompromised individuals, can lead to hesitancy [13]. No prior data have described vaccination coverage among PWLHIV in Italy or evaluated targeted strategies to improve it. Moreover, no study has specifically assessed Tdap vaccination coverage among PWLHIV or the impact of in-hospital vaccination programs for this subgroup. Against this background, we examined how vaccination uptake among PWLHIV changed in our third-level, regional referral center for pregnant women living with HIV following the introduction of in-hospital vaccine administration. This study aims to assess the impact of integrating Tdap vaccination into routine antenatal care within a tertiary referral center for PWLHIV, addressing a significant gap in current Italian data.

## Materials and methods

### Study design and setting

This is a single-center retrospective observational study conducted at the University Hospital Federico II in Naples, Italy. We included all PWLHIV referred to the Regional Referral Centre for HIV in Pregnancy at the University Hospital Federico II in Naples, Italy, between January 2021 and December 2024. Baseline characteristics and pertussis-containing vaccine uptake were retrospectively recorded in a dedicated database. Since January 2023, we have introduced the possibility to vaccinate at the antenatal clinic of the Regional Referral Centre for HIV in Pregnancy. Vaccination is performed by the same obstetricians, midwives, and nurses who follow these patients during antenatal visits. During routine prenatal care, all PWLHIV were offered the pertussis-containing vaccine by medical personnel at monthly or biweekly visits between 27 and 36 weeks’ gestation. Upon patient acceptance, vaccine administration was arranged in coordination with the obstetric staff responsible for administering the immunization. In cases where the patient initially declined the vaccine, dedicated counseling was provided to explain the importance of maternal vaccination for both maternal and neonatal protection. Following this counseling, the opportunity to schedule the vaccination was offered again at a subsequent visit.

This manuscript was prepared in accordance with the STROBE (Strengthening the Reporting of Observational Studies in Epidemiology) statement for observational studies (https://www.strobe-statement.org/checklists/).

### Study population

We included all pregnant women with a confirmed diagnosis of HIV infection who were referred to the Centre between January 1, 2021, and December 31, 2024.

Inclusion criteria were:

- Confirmed HIV infection before or during pregnancy;
- Antenatal follow-up at our Centre for at least two visits after the 26th week of gestation;
- Availability of clinical data and vaccination records.

Exclusion criteria included:

- First or second-trimester pregnancy loss;
- Transfer of care to another facility before 27 weeks.

Included patients were divided into two groups: a *pre-intervention group* (before the introduction of in-hospital vaccination, from January 2021 to December 2022), and a *post-intervention group* (after the introduction of in-hospital vaccination, from January 2023 to December 2024); we compared pertussis-containing vaccine uptake between the two groups.

### Intervention and vaccination protocol

Since January 2023, the Centre has implemented an in-hospital Tdap vaccination program. Before that date, patients were referred to external vaccination centers. From 2023 onward, Tdap vaccination was offered directly within the antenatal clinic during routine obstetric visits between 27 and 36 weeks of gestation, according to Italian national recommendations [6]. PLWH were followed by the same doctors (L.S. and L.L.M.) throughout the pregnancy, and these specialists oversaw counseling and the administration of the pertussis vaccine, ensuring standardized counseling content.

A single-dose prefilled syringe containing a 0.5-mL suspension for injection of the combined tetanus, diphtheria, and acellular pertussis (Tdap) vaccine (Boostrix®, GlaxoSmithKline) has been administered.

After acceptance, a written consent form was signed by each patient, according to local protocol. Vaccination was administered by trained obstetric healthcare providers (midwives, nurses, or physicians) directly involved in the patient’s antenatal care via intramuscular injection into the deltoid muscle of the upper arm.

During each antenatal visit, the obstetric staff discussed the importance of maternal immunization and offered the Tdap vaccine. In case of initial refusal, dedicated counseling was provided by physicians and midwives trained in maternal vaccine communication. The vaccine was then re-offered at the next scheduled visit. Patients were informed about the benefits, timing, and safety of the vaccine, especially for HIV-positive individuals and their infants. Vaccination data were documented in the patient's electronic medical record and confirmed through regional immunization registries when available.

### Study outcomes

This study aimed to evaluate the impact of in-hospital vaccine administration on the uptake of pertussis-containing vaccine among PWLHIV at a tertiary referral center.

The primary outcome was the vaccine coverage rate. Specifically, we compared vaccine coverage rates before and after the implementation of in-hospital vaccination to assess whether this strategy effectively improves maternal immunization in this vulnerable population.

### Data collection

Clinical, demographic, and vaccination data were retrospectively collected from electronic health records using a dedicated database. The following variables were collected:

- Maternal age, parity, ethnicity, drug abuse, smoking, chronic disease, coinfection;
- HIV-related clinical data (CD4 count, HAART regimen, viral load);
- Gestational age at booking and at vaccination;
- Acceptance/refusal of the Tdap vaccine;
- Any adverse effects related to the vaccine.

### Ethics

This study was conducted using anonymized, routinely collected clinical data and did not involve any experimental intervention or deviation from standard care. In accordance with the Italian national [14], Tdap vaccination is explicitly recommended for pregnant women living with HIV as part of standard prenatal care. Our hospital’s direction has introduced and well regulated this procedure since 2023 as part of standard antenatal care for pregnant women referred to the Mother and Child Department of the University Hospital Federico II. We aimed only to compare the vaccine uptake before and after this intervention. All data were managed in accordance with the Declaration of Helsinki and the General Data Protection Regulation (GDPR – EU 2016/679). Written informed consent for the use of anonymized clinical data for research purposes was obtained from all participants. The protocol has been reviewed by the local ethical committee (Campania 3), ensuring compliance with ethical and legal standards for data protection.

### Statistical analyses

Descriptive statistics were used to summarize the data. Continuous variables were expressed as means ± standard deviation (SD) or medians with interquartile ranges (IQR), depending on data distribution assessed with the Shapiro–Wilk test. Categorical variables were presented as absolute numbers and percentages.

Pearson’s Chi-square test or Fisher’s exact test was used for univariate analysis of categorical variables, and ANOVA or the t-test was used for continuous variables. Clopper-Pearson exact 95% confidence intervals were calculated for vaccination proportions. To account for the small sample size and large effect size, Firth’s penalized likelihood logistic regression was performed to estimate the effect of the intervention on vaccination uptake while minimizing bias in the odds ratio estimates.

A two-tailed p-value < 0.05 was considered statistically significant. Statistical analyses were performed using IBM SPSS Statistics version 29.0.1.0 (Chicago, IL, USA).

The post-hoc power analysis was performed using G*Power software (version 3.1.9.7), assuming a two-tailed test with an alpha level of 0.05, and using a Chi-squared test with continuity correction.

## Results

Between January 2021 and December 2024, 41 PWLHIV have been referred to the Regional Referral Centre for HIV in Pregnancy, at University Hospital Federico II, in Naples, Italy. Three patients were excluded from the analysis because they ended up in a first-trimester spontaneous abortion, before they could have access to vaccination. The remaining 38 patients were distributed as follows: 9 in 2021, 6 in 2022, 8 in 2023, and 15 in 2024. Baseline characteristics of included patients are summarized in Table 1. No significant differences have been recorded in baseline characteristics between pre- (2021–2022) and post-intervention (2023–2024). The total Tdap vaccine uptake was 55.3% (21/38) [95% Exact CI 38.3%–71.4%]. In pre-intervention group, 3/15 patients (20% [95% Exact CI 4.3%–48.1%]) received the vaccination, while this percentage rose to 78.3% (18/23) [95% Exact CI 56.3%–92.5%] in the post-intervention group (Fisher’s exact test, *p* < 0.001).

**Table 1:** Baseline characteristics of the included patients, stratified by year of inclusion

Variables	All *n* = 38	Pre-intervention *n* = 15	Post-intervention *n* = 23	*p* value
Maternal age (years)	33.1 ± 6.5	34.4 ± 7.8	32.3 ± 5.6	0.331
Caucasian	26 (68.4)	9 (60)	17 (73.9)	0.367
Body mass index (kg/m^2^)	25.8 ± 5.2	26 ± 5.5	25.7 ± 5.2	0.888
Nulliparity	20 (52.6)	8 (53.3)	12 (52.2)	0.394
Chronic diseases	10 (26.3)	3 (20)	7 (30.4)	0.231
Smoking	2(5.3)	2 (13.3)	0 (0)	0.053
Coinfections	5 (13.2)	3 (20)	2 (13.3)	0.303
Undetectable viral load at term	27(71.1)	11 (84.6)	16 (69.6)	0.317
HAART	37 (97.4)	13(100)	22 (95.7)	0.314
Drug abuse	2 (5.3)	2 (15.4)	–	0.365
Vaccinated Patients	21 (55.3)	3 (20)	18 (78.3)	< 0.001

Based on the observed vaccination rates in the pre-intervention (20.0%) and post-intervention (78.3%) groups, a post hoc power analysis was performed (α = 0.05). The study achieved 93.4% power to detect this difference using the Chi-squared test with continuity correction.

To evaluate the independence of the intervention's effect, a multivariable Firth’s penalized logistic regression was conducted, adjusting for key demographic and clinical factors: age, BMI, parity, and race. The analysis confirmed that the introduction of the in-hospital vaccination program was the primary driver of increased uptake (*aOR* 11.45; 95% CI 2.45–68.32; *p* < 0.001), while age, BMI, parity, and race did not significantly influence vaccination status in this cohort.

During the study period, no adverse effects have been reported among vaccinated women.

No serious adverse effects have been reported after vaccine administration in this population.

## Discussion

This single-center retrospective study evaluated the impact of an in-hospital Tdap vaccination strategy on vaccine uptake among PWLHIV.

We observed a significant increase in Tdap vaccine adherence following the introduction of in-hospital vaccine administration at the antenatal clinic by obstetric healthcare providers. Vaccine uptake rose from 20% in the pre-intervention period (2021–2022) to 78.3% in the post-intervention period (2023–2024).

Vaccine coverage in the pre-intervention group was significantly lower than the Tdap coverage among the general obstetric population. Indeed, the Centers for Disease Control and Prevention (CDC) reported a Tdap coverage rate of around 53.8% in the 2019–2020 season [15], while a multicenter Italian study reported a pertussis vaccine uptake of 59.7% in 2020 [9].

PWLHIV patients are at increased risk of infectious complications during pregnancy due to their immunocompromised status. Moreover, it has been reported that HIV-exposed but uninfected newborns present an increased susceptibility to severe infectious diseases [16]. These newborns have been shown to have a higher susceptibility to respiratory tract infections, including pertussis, and related complications. Therefore, ensuring maternal immunization in this group is a public health priority. A meta-analysis including studies performed in South Africa and Uganda reported an increased risk of Bordetella pertussis infection, hospitalization, and death in HIV-exposed but uninfected newborns [17]. According to these findings, maternal immunization in PWLHIV is particularly important. Moreover, although some studies report lower immunogenicity in PWLHIV with more advanced disease, no risks have been reported in these patients; therefore, maternal immunization remains a standard of care in this population [18, 19]. Nevertheless, as previously reported, vaccination coverage in PWLHIV remains suboptimal compared to the general pregnant population.

There are few studies that specifically examine vaccination coverage among PWLHIV. The limited available literature consistently reports lower vaccination rates in this population than in the general pregnant population [18]; therefore, identifying interventions to improve maternal uptake in this specific category is challenging. Doraivelu et al. observed that HIV-positive status was negatively related to vaccine uptake in the USA. PWLHIV were less likely to be vaccinated against influenza (4.8% *vs* 10,3%) or both influenza and pertussis (39,7% *vs* 43,3%), compared to pregnant women without HIV infection [20]. A recent multicenter cohort study, including 310 pregnancies among women participating in Women’s Health Study (WHS) of the Surveillance Monitoring for ART Toxicities Study of the Pediatric HIV/AIDS Cohort Study, concluded that less than one-third of the included patients underwent the recommended vaccination.:32.6% received Tdap vaccination, 31,6% flu vaccination, and only 22,6% of women received both vaccinations, as recommend [18]. Tourè et al., reported that the vaccine most frequently administered during pregnancy was flu vaccine, followed by COVID-19, and Tdap, among PWLHIV [21], while Tdap is the most common vaccine administered during pregnancy in the general obstetric population.

Several factors contribute to this discrepancy, including barriers to healthcare access, socioeconomic vulnerability, and vaccine hesitancy driven by misinformation or fears about safety and immunogenicity in HIV-positive individuals. Despite the reasons for such low vaccine uptake among PWLHIV, which should be further addressed, previous studies have hypothesized that these patients face more complex psychosocial, socioeconomic, and medical concerns [9, 18]. Migration status has also been reported to impact vaccine uptake among people living with HIV [22, 23]. Moreover, they may encounter barriers to accessing high-quality health care [9]. In our setting, integrating vaccination into routine antenatal care, performed by the same obstetric staff already involved in patient care, resulted in a significant increase in uptake. These findings are consistent with the existing literature, which suggests that vaccine acceptance increases when vaccines are administered directly by trusted providers during routine pregnancy visits [6, 9, 24]. In particular, a recent study reported proactive communication about the benefits of maternal immunization by HIV physicians or prenatal healthcare providers as the main facilitators of vaccine acceptance among PWLHIV, while the main barriers were concerns about the safety of vaccinations and lack of information [25]. Similarly, a small study including 15 PWLHIV and 18 HIV-seronegative pregnant women, and analyzing COVID-19 vaccine coverage, reported that 73% had received the COVID-19 vaccine and that consistency in antenatal care providers, desire to protect the fetus or themselves, and trust in medical care providers were the main drivers [26].

Notably, the European AIDS Clinical Society guidelines do not report recommendations regarding maternal immunization [25].

Our intervention included not only vaccine administration but also a structured counseling process. Several patients initially declined vaccination but later accepted it after targeted education on maternal and neonatal benefits. This underscores the value of repeated offers and personalized communication in promoting vaccine uptake, particularly in populations with greater healthcare-related vulnerabilities.

Despite these encouraging findings, our study has several limitations. First, the sample size was limited due to the specific population and single-center design, which may affect the generalizability of our results. However, a post hoc power analysis for the primary outcome indicated that the study was well powered (93.4%) to detect the significant increase in vaccine uptake observed between the two time periods. Moreover, Firth’s penalized likelihood logistic regression showed that our intervention remained a robust and highly significant predictor of vaccination, even after adjustment for potential clinical and demographic confounders.

Second, although counseling played a critical role in increasing acceptance, we did not systematically assess patients' reasons for initial refusal or explore factors influencing hesitancy through qualitative methods. Moreover, we cannot exclude secular trend bias, such as COVID-19-related hesitancy. We acknowledge that other concurrent, unmeasured factors, such as institutional or public health initiatives, could have contributed to the observed improvement, even if we were unaware of specific prevention campaigns. However, the close temporal association between the implementation of our in-hospital program and the significant rise in vaccination uptake suggests that our intervention was an important driver of this positive outcome. Although we applied the same strategy to all pregnant women, comparison of its effect on the general population was not available, nor was a comparison with a center that did not introduce this policy. As a single-center study conducted in a specialized HIV clinic, the results reflect the specific patient–provider dynamics and institutional protocols of our facility and lack external validation. Therefore, our findings may not be directly generalizable to other settings with different healthcare infrastructures or different levels of patient engagement. Finally, we were unable to assess neonatal outcomes, such as neonatal Tdap antibody titers or the incidence of pertussis among infants, which could further support the clinical impact of improved maternal immunization in this population. However, the study was designed to evaluate the clinical success of the in-hospital vaccination pathway (uptake rates) rather than the biological efficacy of the transferred immunity.

Our findings suggest that integrating maternal Tdap vaccination into routine antenatal care pathways within HIV referral centers can significantly improve vaccine uptake among PWLHIV. This model may be especially effective in contexts where vulnerable populations face fragmented access to external vaccination services. Moreover, it could be applied to other maternal vaccinations, such as the flu vaccine or the COVID-19 vaccine, in order to improve maternal immunization. This is important, given the continued hesitancy reported among pregnant women toward all recommended vaccines.

Not only Tdap but also flu and COVID-19 vaccination coverage are suboptimal during pregnancy. Regarding COVID-19 vaccination, the CDC reported that the percentage of vaccinated pregnant women was below 15% [27]. Moreover, the CDC reported a progressive decline in flu vaccination coverage during pregnancy, from around 60% in the 2019–2020 season to around 38% in the 2024–2025 season [28]. The European Centre for Disease Prevention and Control (ECDC) reported highly variable and generally suboptimal influenza vaccination coverage among pregnant women in Europe, with rates frequently falling below the 75% target set by the European Council. The median coverage was 22% for the 2024–2025 season [29]. Italian data reported even lower vaccination coverage, below 15% [6].

As supported by previous evidence [19], to further enhance maternal immunization, national strategies should promote:

- Universal in-hospital vaccine availability.
- Training of obstetric staff in vaccine counseling;
- Standardized tracking of maternal vaccination across regions;
- Future multicentric studies should explore facilitators and barriers to vaccination in this subgroup.

Moreover, offering vaccinations in multiple settings (e.g., pharmacies, general practices, antenatal clinics, and hospitals) has been recommended as one of the strategies to improve maternal vaccine uptake [24].

Our model could be easily replicated in other tertiary or regional centers, especially where a high volume of antenatal care is provided to women with chronic conditions. Our findings support integrating maternal immunization into standard antenatal protocols and emphasize the need for regional and national health systems to invest in infrastructure that enables in-clinic vaccine delivery.

However, qualitative research is necessary to understand individual factors influencing vaccine hesitancy among PWLHIV, with the goal of designing tailored interventions that address the complex psychosocial needs of this population. Additionally, larger prospective multicenter studies, including data on neonatal outcomes and the impact on future compliance with childhood immunization, are needed to further support our preliminary findings.

## Conclusions

In this single-center retrospective observational study, we observed increased vaccine uptake after the introduction of hospital-based vaccination. Our results support the idea that vaccine availability in the hospital setting and receiving the vaccine from the same obstetric health care provider who performs antenatal visits may be important drivers of maternal adherence to antenatal vaccinations among people who face the daily challenges of a severe chronic condition, such as HIV infection.

Given that the study was conducted at a single site, we acknowledge the limited generalizability of our findings and the need for larger studies to assess facilitators and barriers to vaccination among PWLHIV. However, we believe this intervention contributed meaningfully to improving vaccine coverage in this population and that our model may serve as a framework for improving the uptake of multiple recommended maternal vaccines.

## Data Availability

The data that support the findings of this study are available from the corresponding author upon reasonable request.

## Abbreviations

LD: Linear dichroism
HIV: Human immunodeficiency virus
PWLHIV: Pregnant women living with HIV
Tdap: Tetanus, diphtheria and acellular pertussis

## References

[1] Decker MD, Edwards KM (2021) Pertussis (Whooping Cough). J Infect Dis 224:S310–S320. 10.1093/infdis/jiaa469 34590129 10.1093/infdis/jiaa469 PMC8482022

[2] Perin J, Mulick A, Yeung D, Villavicencio F, Lopez G, Strong KL et al (2022) Global, regional, and national causes of under-5 mortality in 2000–19: an updated systematic analysis with implications for the Sustainable Development Goals. Lancet Child Adolesc Health 6:106–115. 10.1016/S2352-4642(21)00311-4 34800370 10.1016/S2352-4642(21)00311-4 PMC8786667

[3] Fiasca F, Necozione S, Mattei A (2021) Pertussis in Italy: how to protect the “unprotectable”? Hum Vaccin Immunother 17:1136–1141. 10.1080/21645515.2020.1806673 33121322 10.1080/21645515.2020.1806673 PMC8018419

[4] De Weerdt L, Herzog SA, Van Damme P, Maertens K (2024) Timing of pertussis vaccination during pregnancy: evidence and implementation – a systematic review. Vaccine 42:126152. 10.1016/j.vaccine.2024.07.053 39088988 10.1016/j.vaccine.2024.07.053

[5] Vygen-Bonnet S, Hellenbrand W, Garbe E, von Kries R, Bogdan C, Heininger U et al (2020) Safety and effectiveness of acellular pertussis vaccination during pregnancy: a systematic review. BMC Infect Dis 20:136. 10.1186/s12879-020-4824-3 32054444 10.1186/s12879-020-4824-3 PMC7020352

[6] Vilca LM, Sarno L, Cesari E, Vidiri A, Antonazzo P, Ravennati F et al (2021) Differences between influenza and pertussis vaccination uptake in pregnancy: a multi-center survey study in Italy. Eur J Public Health 31:1150–1157. 10.1093/eurpub/ckab095 34580721 10.1093/eurpub/ckab095

[7] Kahn KE, Black CL, Ding H, Williams WW, Lu P-J, Fiebelkorn AP et al (2018) Influenza and Tdap vaccination coverage among pregnant women — United States, April 2018. MMWR Morb Mortal Wkly Rep 67:1055–1059. 10.15585/mmwr.mm6738a3 30260946 10.15585/mmwr.mm6738a3 PMC6188122

[8] Khalil A, Samara A, Campbell H, Ladhani SN, Amirthalingam G (2024) Recent increase in infant pertussis cases in Europe and the critical importance of antenatal immunizations: we must do better…now. Int J Infect Dis 146:107148. 10.1016/j.ijid.2024.107148 38960028 10.1016/j.ijid.2024.107148

[9] Vilca LM, Sarno L, Passoni D, Antonazzo P, Pellegrini E, Guida M et al (2024) Impact of the COVID-19 pandemic on prenatal care utilization among Italian and immigrant pregnant women: a multicenter survey. Int J Public Health. 10.3389/ijph.2024.1606289 38440081 10.3389/ijph.2024.1606289 PMC10910076

[10] Poeta M, Moracas C, Albano C, Petrarca L, Maglione M, Pierri L et al (2024) Pertussis outbreak in neonates and young infants across Italy, January to May 2024: implications for vaccination strategies. Eurosurveillance. 10.2807/1560-7917.ES.2024.29.23.2400301 38847118 10.2807/1560-7917.ES.2024.29.23.2400301 PMC11158011

[11] Cremer M, Kaempfen S, Lapaire O, Hoesli IM, Heininger U (2024) Interventional study to improve pertussis and influenza vaccination uptake in pregnant women. Eur J Obstet Gynecol Reprod Biol 295:201–209. 10.1016/j.ejogrb.2024.02.019 38367393 10.1016/j.ejogrb.2024.02.019

[12] Razai MS, Berendes S, Nagraj S, Oakeshott P, Khalil A (2024) As whooping cough rates rise, we urgently need to increase maternal vaccination rates. BMJ. 10.1136/bmj.q1289 38871363 10.1136/bmj.q1289

[13] Razzaghi H, Kahn KE, Calhoun K, Garacci E, Skoff TH, Ellington SR et al (2023) Influenza, Tdap, and COVID-19 vaccination coverage and hesitancy among pregnant women — United States, April 2023. MMWR Morb Mortal Wkly Rep 72:1065–1071. 10.15585/mmwr.mm7239a4 37768879 10.15585/mmwr.mm7239a4 PMC10545430

[14] Presidenza del Consiglio dei Ministri Conferenza permanente per i rapporti tra lo stato le regioni e le province autonome. Piano nazionale di prevenzione vaccinale (PNPV) 2023–2025. Gazzetta Ufficiale 2023.

[15] Centers for Disease Control and Prevention. Flu, Tdap, and COVID-19 Vaccination Coverage Among Pregnant Women – FluVaxView. FluVaxView 2024.

[16] Dauby N, Gagneux-Brunon A, Martin C, Mussi-Pinhata MM, Goetghebuer T (2024) Maternal immunization in women living with HIV. AIDS 38:137–144. 10.1097/QAD.0000000000003758 38116721 10.1097/QAD.0000000000003758

[17] Muloiwa R, Kagina BM, Engel ME, Hussey GD (2020) The burden of laboratory-confirmed pertussis in low- and middle-income countries since the inception of the Expanded Programme on Immunisation (EPI) in 1974: a systematic review and meta-analysis. BMC Med 18:233. 10.1186/s12916-020-01699-3 32854714 10.1186/s12916-020-01699-3 PMC7453720

[18] Berhie S, Kacanek D, Lee J, Jao J, Powis K, Salomon L et al (2024) Routine vaccination during pregnancy among people living with HIV in the United States. JAMA Netw Open 7:e249531. 10.1001/jamanetworkopen.2024.9531 38696165 10.1001/jamanetworkopen.2024.9531 PMC11066702

[19] El Chaer F, El Sahly HM (2019) Vaccination in the adult patient infected with HIV: a review of vaccine efficacy and immunogenicity. Am J Med 132:437–446. 10.1016/j.amjmed.2018.12.011 30611828 10.1016/j.amjmed.2018.12.011

[20] Doraivelu K, Boulet SL, Biswas HH, Adams JC, Haddad LB, Jamieson DJ (2019) Predictors of tetanus, diphtheria, acellular pertussis and influenza vaccination during pregnancy among full-term deliveries in a medically underserved population. Vaccine 37:6054–6059. 10.1016/j.vaccine.2019.08.044 31471152 10.1016/j.vaccine.2019.08.044

[21] Touré Y, Martin C, Necsoi C, Delforge M, Konopnicki D, Dauby N (2025) Experience of maternal immunization among women living with HIV in Belgium: a mixed-methods study. Prev Med Rep. 10.1016/j.pmedr.2025.103107 40474991 10.1016/j.pmedr.2025.103107 PMC12140069

[22] Grabmeier-Pfistershammer K, Herkner H, Touzeau-Roemer V, Rieger A, Burgmann H, Poeppl W (2015) Low tetanus, diphtheria and acellular pertussis (Tdap) vaccination coverage among HIV infected individuals in Austria. Vaccine 33:3929–3932. 10.1016/j.vaccine.2015.06.056 26102535 10.1016/j.vaccine.2015.06.056

[23] Gobert C, Van Hauwermeiren C, Quoidbach C, Reschner A, Necsoi C, Benslimane A et al (2021) Tetanus seroprotection in people living with HIV: risk factors for seronegativity, evaluation of medical history and a rapid dipstick test. Vaccine 39:1963–1967. 10.1016/j.vaccine.2021.02.062 33715902 10.1016/j.vaccine.2021.02.062

[24] Razai MS, Mansour R, Goldsmith L, Freeman S, Mason-Apps C, Ravindran P et al (2023) Interventions to increase vaccination against COVID-19, influenza and pertussis during pregnancy: a systematic review and meta-analysis. J Travel Med. 10.1093/jtm/taad138 37934788 10.1093/jtm/taad138 PMC10755181

[25] Touré Y, Martin C, Necsoi C, Delforge M, Konopnicki D, Dauby N (2025) Experience of maternal immunization among women living with HIV in Belgium: a mixed-methods study. Prev Med Rep 54:103107. 10.1016/j.pmedr.2025.103107 40474991 10.1016/j.pmedr.2025.103107 PMC12140069

[26] Berhie SH, DiTosto JD, Cabatu M, Jao J, Cameron K, Kacanek D et al (2023) Drivers and deterrents of COVID-19 vaccination among a diverse, pregnant population with and without HIV. Am J Obstet Gynecol 228:S571–S572. 10.1016/j.ajog.2022.11.970

[27] Centers for Disease Control and Prevention. Pregnant Women COVID-19 Vaccination Coverage 2025. https://www.cdc.gov/covidvaxview/weekly-dashboard/pregnant-women-vaccination.html.

[28] Centers for Disease Control and Prevention. 2023–24 Influenza Vaccination Coverage, Pregnant Women at a glance 2025. https://www.cdc.gov/fluvaxview/dashboard/2023-24-pregnant-women-coverage.html.

[29] European Centre for Disease Prevention and Control. Survey report on national seasonal influenza vaccination recommendations and coverage rates in EU/EEA countries, 2024/25 Surveillance and monitoring 2025. https://www.ecdc.europa.eu/en/publications-data/survey-rep.

